# Effect of angiotensin II on irradiation exacerbated decompression sickness

**DOI:** 10.1038/s41598-023-38752-z

**Published:** 2023-07-19

**Authors:** Jie-Fu Fan, Yang-Kai Wang, Min Liu, Guang-Sheng Liu, Tian-Jiao Min, Rui-Yong Chen, Ying He

**Affiliations:** Naval Medical Center of PLA, Naval Medical University (Second Military Medical University), Shanghai, China

**Keywords:** Diseases, Medical research, Pathogenesis

## Abstract

In some complicated situations, decompression sickness (DCS) combined with other injuries, such as irradiation, will seriously endanger life safety. However, it is still unclear whether irradiation will increase the incidence of DCS. This study was designed to investigate the damage effects of irradiation on decompression injury and the underlying mechanism. Sprague–Dawley rats were exposed to irradiation followed by hyperbaric decompressing and the mortality and decompression symptoms were observed. Lung tissue and bronchoalveolar lavage fluid were collected to detect the lung lesion, inflammation response, activity of the angiotensin system, oxidative stress, and relative signal pathway by multiple methods, including Q-PCR, western blot, and ELISA. As a result, pre-exposure to radiation significantly exacerbated disease outcomes and lung lesions of DCS. Mechanically, the up-regulation of angiotensin-converting enzyme expression and angiotensin II levels was responsible for the exacerbated DCS and lung lesions caused by predisposing irradiation exposure. Oxidative stress and PI3K/AKT signal pathway activation in pulmonary tissue were enhanced after irradiation plus decompression treatment. In conclusion, our results suggested that irradiation could exacerbate lung injury and the outcomes of DCS by activating the angiotensin system, which included eliciting oxidative stress and activation of the PI3K/AKT signal pathway.

## Introduction

Decompression sickness (DCS) is a syndrome consisting of a constellation of symptoms following an unsafe decompression process after hyperbaric exposure^1^, such as survival escape after a disabled submarine. In specific conditions, like a disabled nuclear submarine accident, the survivors could suffer radiation exposure before the fast buoyancy ascent escape. However, the impact of predisposing radiation exposure on the DCS has not been evaluated. More importantly, there is a lack of practical prevention and treatment strategy for such cases. As for common diving, measures to reduce DCS include hyperbaric training, predive vibration, and preoxygenation, which have high requirements for space and equipment^2,3^. In recent studies, the pharmacological treatment and prevention of DCS is the focus of research. Thus, to better save life and minimize damage in a disabled submarine accident, it is essential to identify the impact of radiation exposure on the safety of fast buoyancy ascent escape, the underlying mechanism, and the potential target for pharmacological treatment.

In DCS, arterial gas embolism (AGE) and cardiopulmonary distress are the most contributing factors to death^1,4^. Mechanically, supersaturated gas forms microbubbles and convergences to the pulmonary capillary network through the venous system, causing pulmonary capillaries damage and a myriad of vascular reactions. Expanding gas further stretches and ruptures alveolar arteries, leading to pulmonary barotrauma and even AGE^4,5^. It has been reported that robust lung function and the avoidance of pulmonary inflammation could reduce the risk of DCS^1,6^. Moreover, the lung also showed considerable sensitivity to irradiation^7^. Acute irradiation could elicit vascular inflammation and reactions^8,9^. Hence, an essential question arises about the impact of pre-exposure to radiation on lung injury in DCS.

Vascular dysfunction and inflammation are critical pathogenic processes in the development of irradiation and DCS, respectively^10–13^. Identifying the factor which plays a role in these two biological events may be critical to demonstrating the mechanism underlying exacerbated DCS after radiation exposure. Angiotensin II (Ang II) is a peptide hormone well-known for its vasoactive and pro-inflammatory properties^14–16^. Angiotensin-converting enzyme (ACE), the critical enzyme of Ang II production, is abundant in pulmonary vessels^17^. In radiation exposure, local Ang II has been demonstrated to mediate multiple damage responses in radiation pneumonitis^18,19^. Moreover, it has been reported that captopril, an ACE inhibitor, could significantly ameliorate endothelial dysfunction caused by irradiation^20^. In DCS patients, the plasma level of Ang II was also increased^21,22^. The above studies highlighted the importance of Ang II in vascular damage and inflammation in irradiation and DCS. Hence, we hypothesized that Ang II takes part in the impact of irradiation pre-exposure on lung injury in DCS.

Based on the above, we hypothesized that radiation exposure could increase the risk of DCS through the activation of the angiotensin system and exacerbate lung injury. Therefore, in the present study, we detected lung damage and the activation of the angiotensin system in the injury of radiation plus decompression. Furthermore, we explored the mechanism underlying angiotensin system activation, identifying the effect of oxidative stress and the associated signal pathway.

## Methods

### Animals

Seven-week-old male normotensive Sprague–Dawley (SD) rats were supplied by Shanghai Lingchang Biotechnology Co., Ltd, China. Rats were housed in a temperature-controlled room and kept on a 12:12 h light–dark cycle with free access to food and water. All procedures in this study were approved by the Committee of Animal Care and Use, at Naval Medical University. All operations were conducted following the Guide for the Care and Use of Laboratory Animals published by the US National Institutes of Health (NIH publication No. 85–23, revised 1985) and carried out in compliance with the ARRIVE guidelines (http://www.nc3rs.org.uk/page.asp?id=1357). A total of 100 animals were randomly divided into five groups: blank control (CT, n = 40), irradiation only (IR, n = 20), decompression only (DC, n = 20), irradiation plus decompression (ID, n = 20), irradiation/decompression plus normal saline pretreatment (ID + NS), irradiation/decompression plus enalapril pretreatment (ID + EL, n = 20), and irradiation/decompression plus losartan pretreatment (ID + LS, n = 20).

### Irradiation and hyperbaric exposure protocol

The irradiation exposure was performed in the Naval Medical Center of PLA. Rats were individually placed in cages with the whole body exposed to irradiation. ^60^Co γ-rays at a dose of 4 Gy irradiation (a dose rate of 0.65 Gy/min) were performed for 370 s in each rat, and the distance to the source was approximately 5.13 m.

Hyperbaric exposure was using the animal hyperbaric chamber in the Naval Specialty Medical Center. The pressure of 6.7 ATA is established with high-pressure air and maintained for 45 min. Keep ventilation in the animal cabin during the period, then surface at a constant speed for 30 ± 5 s.

In the ID group, rats were exposed to radiation first and then to the decompression treatment one hour later. The irradiation and decompression treatments, respectively, were the same as the single-treatment group. In the ID + EL group and ID + LS group, intraperitoneal injections of enalapril (100 mg/kg) and losartan (100 mg/kg) were treated 30 min before irradiation treatment.

### Observation of DCS symptoms

Immediately after decompression treatment, the rats were transferred to the animal cages. The behavioural performance of the rats was observed and recorded for 30 min. DCS was diagnosed by three observers who were unaware of the treatment. According to the evaluation criteria of decompression sickness^23,24^, the rats were diagnosed as DCS if any one of the symptoms of scratching, sluggishness, tachypnea or dyspnea, cyanosis, paralysis, convulsions, and death occurred after treatment. Severe DCS was defined as having at least one symptom, including paralysis, dyspnea, convulsions, and death. The onset time and symptoms of rats were recorded in detail.

### Tissue harvesting and histopathological evaluation of lung

Lung tissues were removed from the rats in each group after symptom observation for 30 min after decompression treatment. An enzyme-linked immunosorbent assay (ELISA) was used to detect ACE enzyme activity, Ang II concentration, and reactive oxygen species (ROS) level in collected lung tissue to the protocol (ELISA Kit for ACE, HB1258-Ra, Henyuan Biotechnology, China), (ELISA Kit for Ang II, HB193-Ra, Henyuan Biotechnology, China), (Reactive Oxygen Species Assay Kit, P-846-SH, Henyuan Biotechnology, China). For histological studies, the excised lung was instilled with 10% of buffered formalin at a regular temperature and immersed in buffered formalin overnight. The tissues were processed for conventional paraffin histology, section, stained with hematoxylin and eosin (H&E) and examined by light microscopy. Lung injury was measured based on the findings in 10 randomly selected high-power fields (*200) for each tissue slide. Oedema, alveolar and interstitial inflammation and hemorrhage, atelectasis, necrosis, and hyaline membrane formation were separately scored on a 0- to 4-point scale: 0, no injury; 1, injury in 25% of the field; 2, injury in 50% of the field; 3, injury in 75% of the field; and 4, injury throughout the field; the final lung injury score was obtained by adding these scores^25^.

### Lung wet-to-dry weight ratio assay

The upper lobe of the right lung was collected after sacrifice and the wet weight was accurately measured with a microbalance. Then the tissue was placed in an 80 °C oven for 72 h and reweighed for dry weight detection. Subsequently, the lung wet-to-dry ratio (W/D) was calculated according to the formula W/D = wet weight (g)/dry weight (g)^26^.

### Bronchoalveolar lavage fluid analysis

Rats were anaesthetized with urethane (800 mg/kg i.p.) and a-chloralose (40 mg/kg i.p.) after symptom observation. Bronchoalveolar lavage was then performed as in previous studies^27,28^. Briefly, bronchoalveolar lavage fluid (BALF) was obtained using a syringe filled with 2 ml of 0.9% saline, which was repeated three times, and the total volume recovered was more than 80%. The lavage fluid was centrifuged at 1200 rpm for 10 min. An ELISA was used to detect interleukin-1β (IL-1β), IL-6, TNF-α, and Ang II concentration in collected BALF to the protocol (ELISA Kit for IL-1β, HS972-Ra, Henyuan Biotechnology, China), (ELISA Kit for IL-6, HS959-Ra, Henyuan Biotechnology, China), (ELISA Kit for TNF-α, HS044-Ra, Henyuan Biotechnology, China).

### Quantitative real-time PCR analysis (RT-qPCR)

Total RNA was extracted using the Trizol reagents (Invitrogen, USA) in compliance with the manufacturer’s protocol and then reverse-transcribed into cDNA with the PrimeScript RT reagent kit (Takara, China). 0.5 μg RNA was used as a template. Quantitative real-time PCR was carried out in a LightCycler96 real-time PCR detection system (Roche, Switzerland) using the SYBR Green kit (Takara, China). The primer sequences are shown in Table 1.

**Table 1:** The primer sequences.

Gene		Primer sequences
Rat GAPDH	Forward	5′- GCAAGTTCAACGGCACAG -3′
Rat GAPDH	Reverse	5′- GCCAGTAGACTCCACGACAT -3′
Rat IL-1β	Forward	5′- GGGATGATGACGACCTGCT-3′
Rat IL-1β	Reverse	5′- CCACTTGTTGGCTTATGTTCTG -3′
Rat IL-6	Forward	5′-ACTTCCAGCCAGTTGCCTTCTTG -3′
Rat IL-6	Reverse	5′-TGGTCTGTTGTGGGTGGTATCCTC -3′
Rat TNF-α	Forward	5′- AAAGGACACCATGAGCACGGAAAG-3′
Rat TNF-α	Reverse	5′- CGCCACGAGCAGGAATGAGAAG-3′
Rat ACE	Forward	5′- GTTGCCAATGACATAGAAAGT -3′
Rat ACE	Reverse	5′- CACCAGTCGTAGTTGTAGCG -3′
Rat AT1R	Forward	5′- CCATCGTCCACCCAATGAAG -3′
Rat AT1R	Reverse	5′- TTGGTGTTCTCGATGAAGTATAC -3′
Rat NOX2	Forward	5′- GGTCCCATGTTCCTGTATCTGTGTG -3′
Rat NOX2	Reverse	5′- TGTCCCACCTCCATCCTGAATCC -3′

### Western blot analysis

As previously described^29^, Western blot analysis was performed for determining the protein expression levels in collected lung tissue. The protein concentration was measured and protein (30 μg) was run on a 7.5% or 12% SDS-PAGE gel and then transferred to a polyvinylidene fluoride membrane (Millipore, Germany). The membrane was probed with primary antibodies, NOX2 (381293, Zen Bio), p-Akt (#4060, Cell Signaling Technology, USA) and Akt (#4691, Cell Signaling Technology, USA). The levels of target proteins were normalized to β-actin (380624, Zen Bio, China) which served as a loading control.

### Data analysis

Experimental data were analyzed with GraphPad Prism software 8.0 (San Diego, CA, USA) and shown as means ± SEM. The chi-square test was used to compare the disease outcomes between different groups. Comparisons between multiple groups were analyzed by one-way ANOVA, and the followed Tukey’s multiple comparisons tests were used to analyze the difference between each group in the multi-group comparisons. Differences were considered to be significant at *P* < 0.05.

## Results

### Irradiation exacerbated the disease outcomes and lung injury in DCS

**Figure 1:**
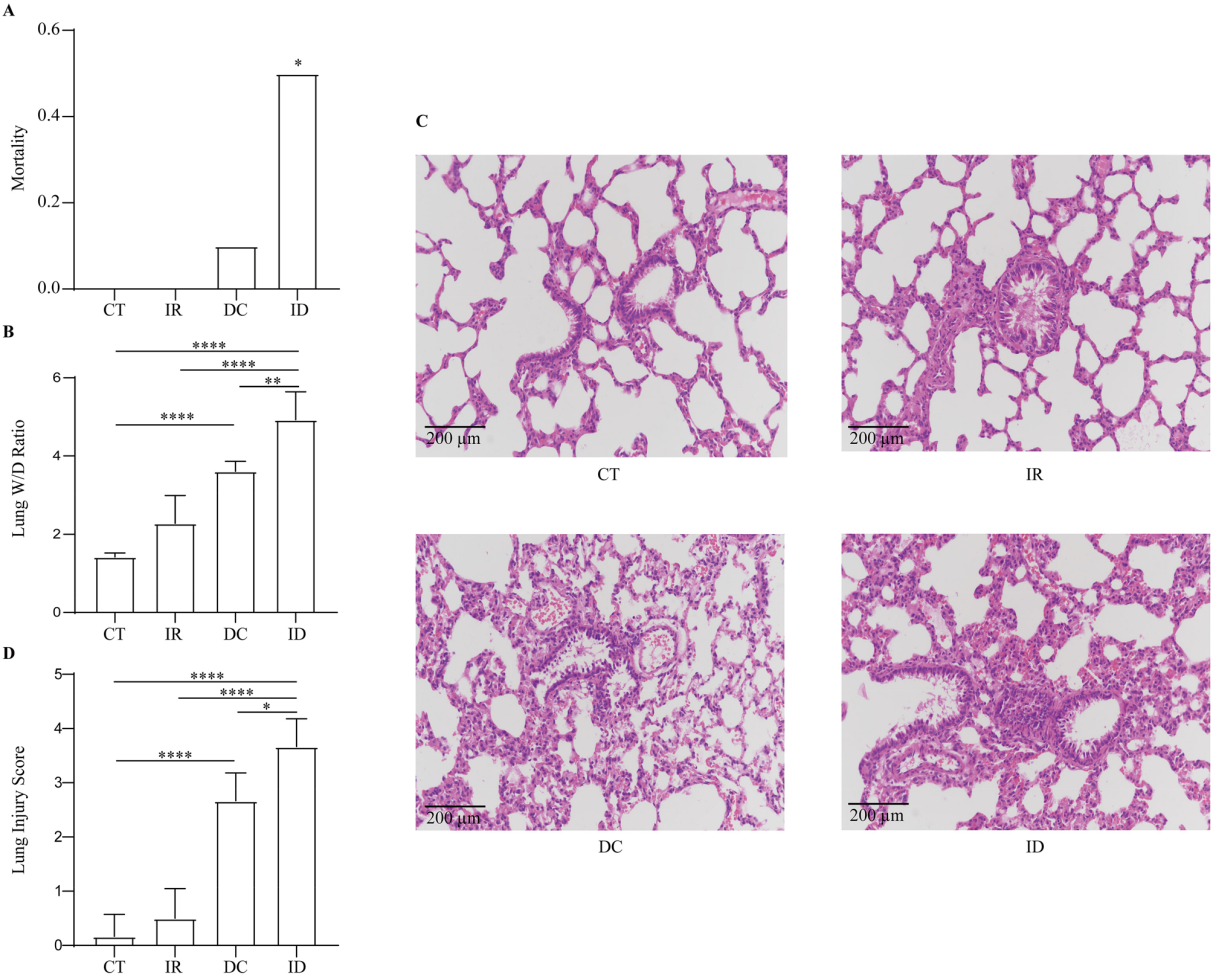
Disease outcomes and lung injury in the CT, IR, DC, and ID groups. The mortality (**A**) and Lung W/D ratio (**B**) after different exposure treatments. Representative images of lung tissue sections (**C**) and the lung injury score (**D**) in each group. Values are mean ± SE; n = 20 in each group of (**A**); n = 5 in each group of (**B**); n = 6 in each group of (**C**) and (**D**). *P < 0.05, **P < 0.01; ***P < 0.001; ****P < 0.0001.

We observed the DSC-related symptoms after irradiation, decompression, and irradiation plus decompression injury in SD rats for 30 min. 14 (70%) rats in the DC group and 18 (90%) rats in the ID group were diagnosed as DCS for having at least one specific symptom (Table 2). Decompression treatment induced 2 (10.0%) deaths out of 20 rats. All rats of the IR group survived until the time of sacrifice. Notably, in the ID group, 10 (50.0%) rats expired, showing significantly higher mortality than in other groups (n = 20; *P* < 0.05) (Fig. 1A).

**Table 2:** DSC related symptoms observed in CT, IR, DC, and ID groups within 30 min.

DCS related symptoms	CT	IR	DC	ID
Scratching	2	17	11	13
Sluggishness	2	0	4	4
Tachypnea	0	0	2	5
Dyspnea	0	0	3	8
Cyanosis	0	0	6	11
Paralysis	0	0	1	5
Convulsions	0	0	1	3
Death	0	0	2	10
Morbidity	–	–	14 (70.0%)	18 (90.0%)
Mortality	0 (0.0%)	0 (0.0%)	2 (10.0%)	10 (50.0%)

We further detected lung injury induced by all kinds of treatments. The Lung W/D ratio was elevated after decompression (3.607 ± 0.255) treatment, while the change was not significant after irradiation treatment (2.283 ± 0.706) (Fig. 1B). Notably, the W/D ratio was significantly higher in the ID group (4.926 ± 0.718) than in the IR and DC groups (Fig. 1B). Histopathologic changes assessed by H&E-stained lung sections were shown in Fig. 1C. The lung lesion caused by irradiation was not significant. Parenchymal and vascular changes were observed in the DC group, including pulmonary artery damage, diffuse alveolar damage, and edema. In the ID group, the lungs exhibited more severe changes in increased alveolar septal thickening, interstitial edema, and vascular congestion as well as substantial neutrophil infiltration in the interstitium and diffuse alveolar hemorrhage and collapse. The lung lesion was quantified by the lung injury score, showing increased scores in the DC group (2.667 ± 0.516) compared with the CT group (0.167 ± 0.408), and the highest score in the ID group (3.833 ± 0.753) (Fig. 1D).

### Irradiation exacerbated pulmonary inflammation in DCS

**Figure 2:**
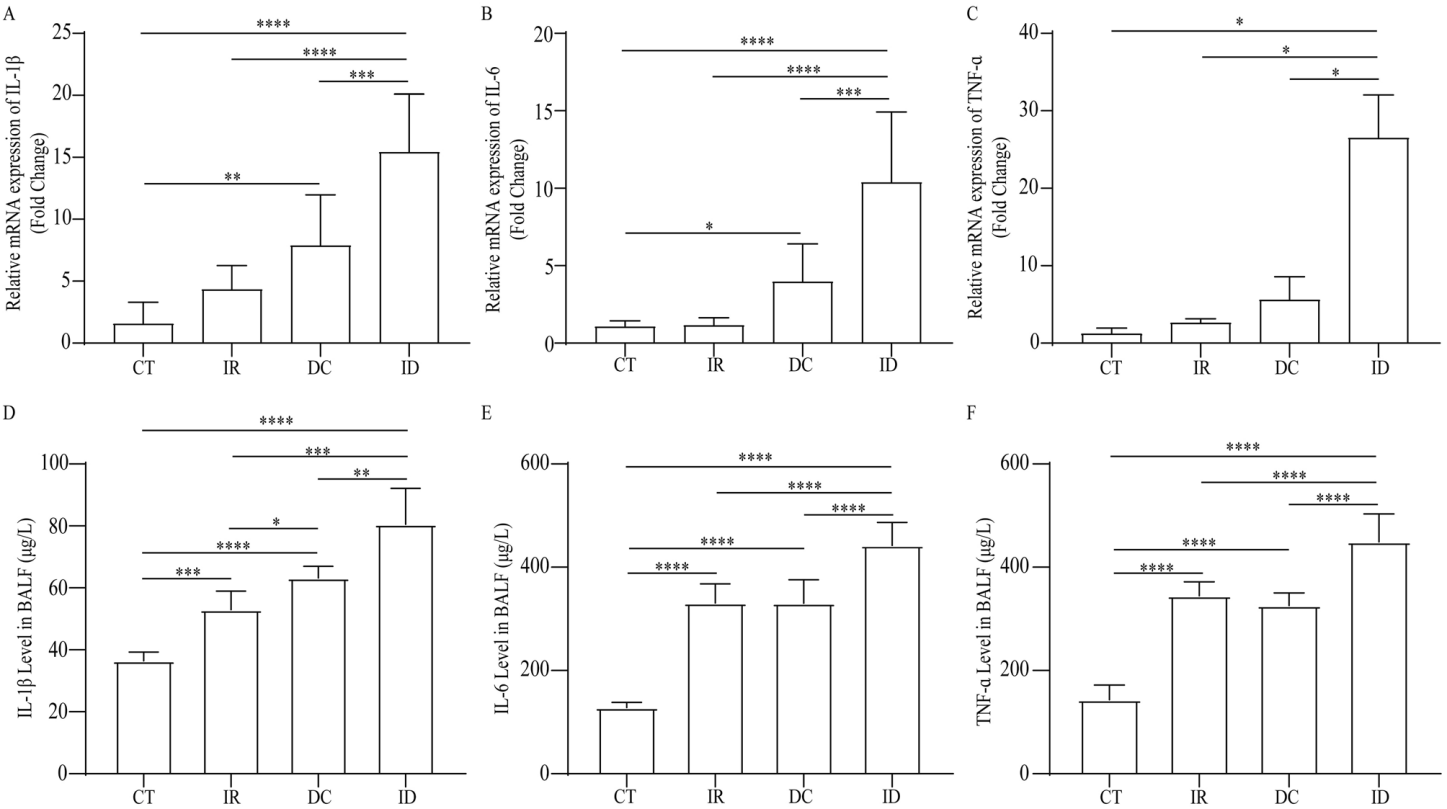
The inflammation in the lungs after irradiation, decompression, and irradiation plus decompression injury. The mRNA levels of IL-1β (**A**), IL-6 (**B**), and TNF-α (**C**) in each group. The levels of IL-1β (**D**), IL-6 (**E**), and TNF-α (**F**) in BALF after different exposure treatments. Values are mean ± SE; n = 5 in each group. *P < 0.05, **P < 0.01; ***P < 0.001; ****P < 0.0001.

The inflammation response generated by irradiation, decompression, and irradiation plus decompression injuries was detected. In the lungs, mRNA levels of IL-1β, IL-6, and TNF-α were elevated after irradiation or decompression treatment, while it was significantly higher in the ID group than in the IR and DC groups (n = 5; *P* < 0.05) (Fig. 2A–C). Concentrations of IL-1β, IL-6, and TNF-α in the BALF showed similar trends, which were significantly higher in the ID group than in the IR and DC ones (n = 5; *P* < 0.05) (Fig. 2D–F).

### Irradiation promoted the up-regulation of Ang II synthesis in DCS

**Figure 3:**
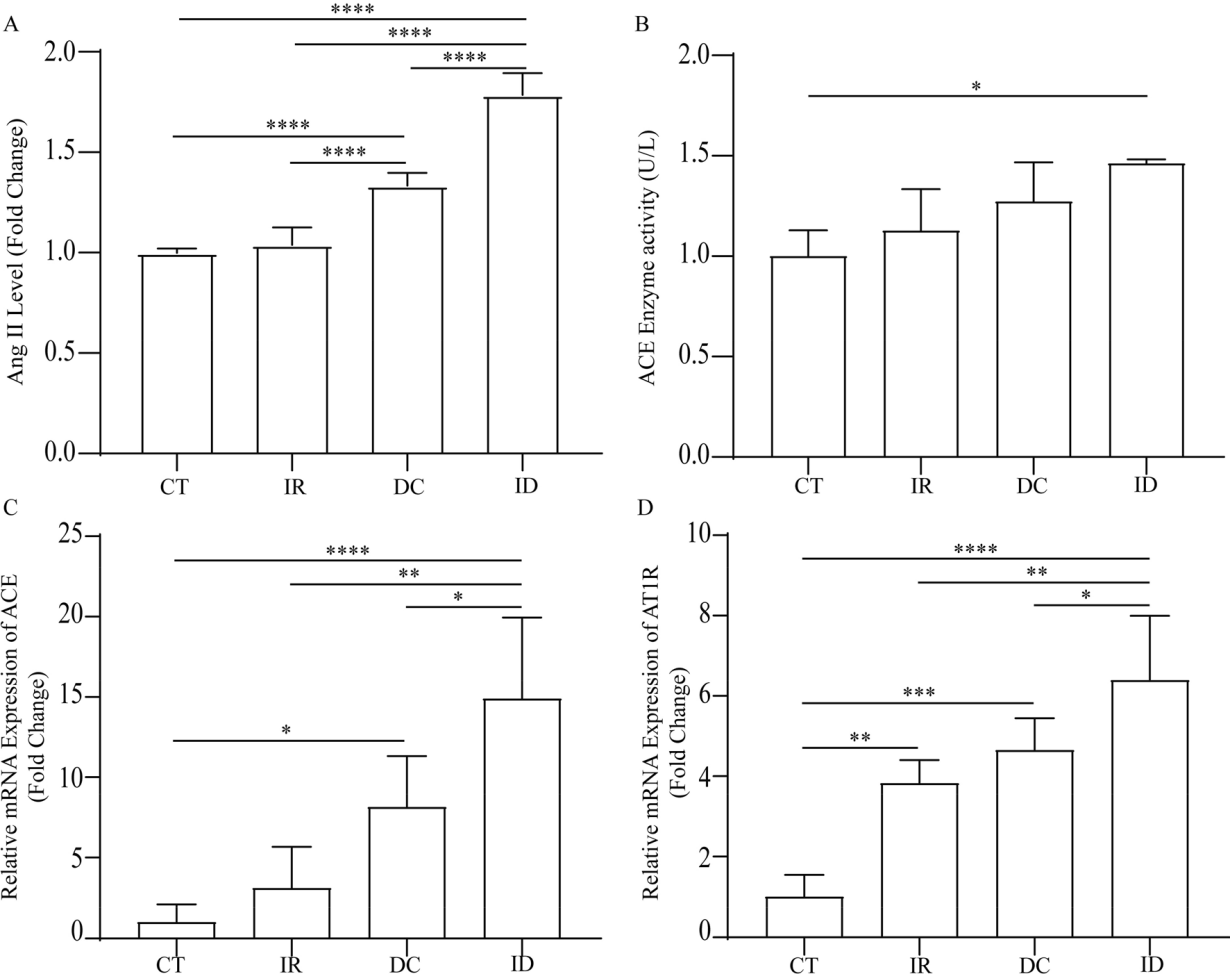
The impact of irradiation, decompression, and irradiation plus decompression injury on the angiotensin system. The BALF Ang II level in each group (**A**). The ACE enzyme activity (**B**) and mRNA levels of ACE (**C**) and AT1R (**D**) in the lungs after different exposure treatments. Values are mean ± SE; n = 5 in each group. *P < 0.05, **P < 0.01; ***P < 0.001; ****P < 0.0001.

To explore the mechanism underlying the exacerbated DCS after irradiation, we focused on the angiotensin system. Irradiation made no difference to the level of Ang II in the lung, yet it was elevated in the IR and ID group compared with the CT group (Fig. 3A). Similarly, we found significant increases in the ACE mRNA level and enzyme activity in the lung tissue of the ID group compared with the CT group, which is responsible for the increased level of Ang II (Fig. 3B, C). We further detected the change in the angiotensin 1 receptor (AT_1_R), the critical receptor mediating the effects of Ang II. The AT_1_R mRNA level was elevated after irradiation or decompression treatment, while it was significantly higher in the ID group than in the IR and DC groups (Fig. 3D).

### ACEI blunted the exacerbated lung damage by irradiation in DCS

**Figure 4:**
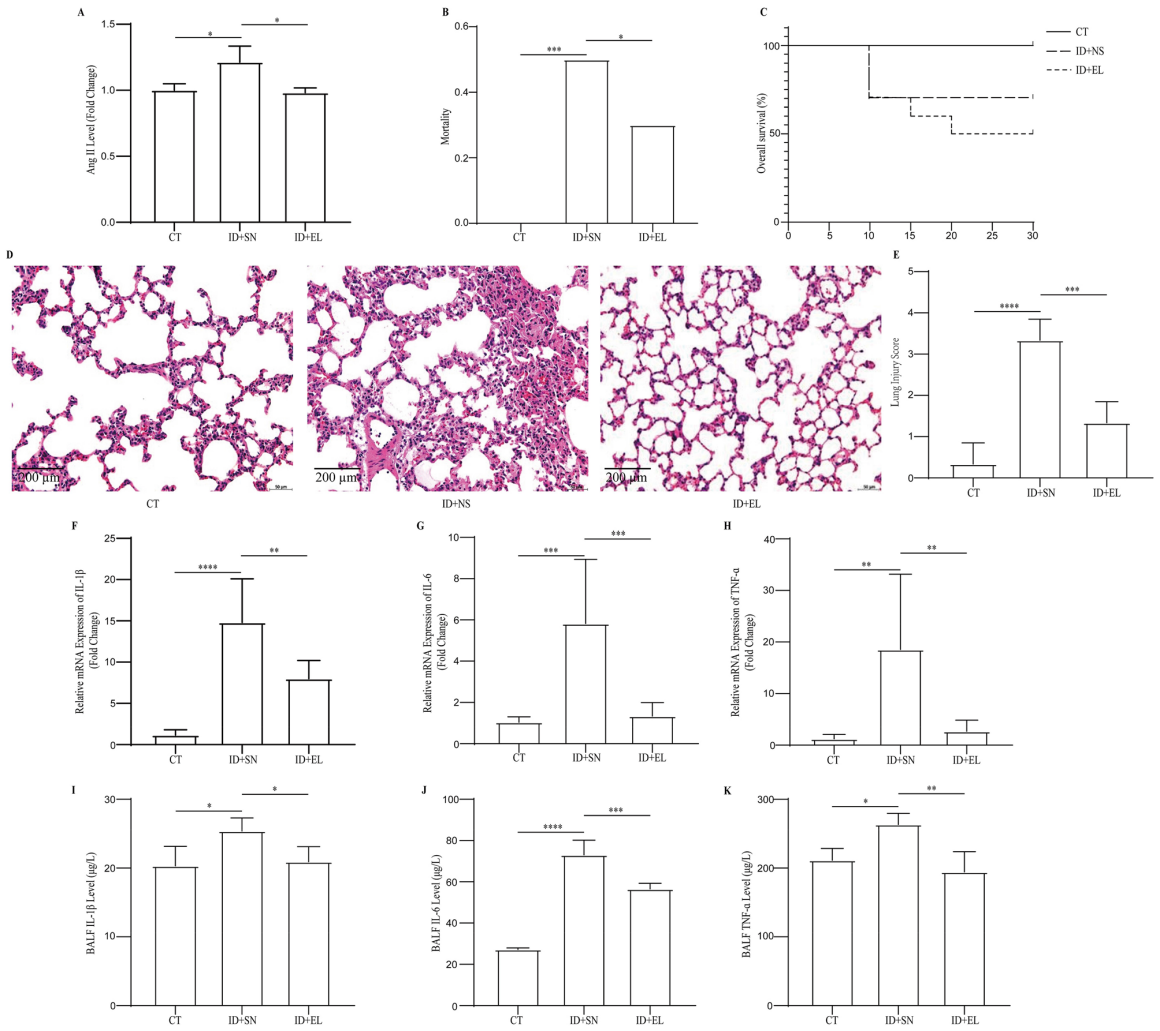
ACEI blunted the exacerbated lung damage by irradiation in DCS. The BALF Ang II level after irradiation plus decompression injury with or without enalapril treatment (**A**). The overall mortality (**B**) and survival curve in each group (**C**). Representative images of lung tissue sections (**D**) and the Lung injury score (**E**) in each group. The mRNA levels of IL-1β (**F**), IL-6 (**G**), and TNF-α (**H**) in each group. The levels of IL-1β (I), IL-6 (**J**), and TNF-α (K) in BALF after irradiation plus decompression injury with or without enalapril treatment. Values are mean ± SE; n = 5 in each group of (**A**), (**F**), (**G**), (**H**), (**I**), (**J**), and (**K**); n = 20 in each group of (**B**) and (**C**); n = 6 in each group of (**D**) and (**E**). *P < 0.05, **P < 0.01; ***P < 0.001; ****P < 0.0001.

To validate the role of Ang II in the exacerbated DCS after irradiation, we pre-treated rats with enalapril, an angiotensin-converting enzyme inhibitor (ACEI), before irradiation and decompression injury. The significant decrease of Ang II level in the lung showed the effectiveness of enalapril treatment (1.213 ± 0.054 vs. 0.982 ± 0.016 ng/L; n = 5; *P* < 0.05) (Fig. 4A). Notably, the mortality in ID + EL group was significantly lower than in ID + NS group (n = 20; *P* < 0.05) (Fig. 4B, C). Moreover, 17 (85.0%) rats were diagnosed as DCS and 9 (45%) of them expired in the ID + LS group. The details of mortality, morbidity and observation of the DCS symptoms were shown in Table 3. The histopathological section of the lungs indicated the protective effect of pre-enalapril treatment (Fig. 4D, E). As the pathologic changes, including infiltration of inflammatory cells and red blood cells, were not prominent in the alveolar space after enalapril pre-treatment. Moreover, we found significant alleviation in alveolar septal thickening and interstitial edema in rats of ID + EL. The irradiation plus decompression injury-induced increases in inflammation indicators, including the level of IL-1β, IL-6, and TNF-α in the lung tissue and BALF, were significantly blunted in enalapril-treated rats (Fig. 4F–K).

**Table 3:** DSC related symptoms observed in CT, ID + NS, ID + EL, and ID + LS groups within 30 min.

DCS Related Symptoms	CT	ID + NS	ID + EL	ID + LS
Scratching	2	15	11	14
Sluggishness	3	3	3	3
Tachypnea	0	5	2	6
Dyspnea	0	8	3	5
Cyanosis	0	9	6	5
Paralysis	0	5	2	4
Convulsions	0	3	2	3
Death	0	10	6	9
Morbidity	–	19 (95.0%)	16 (80.0%)	17 (85.0%)
Mortality	0 (0.0%)	10 (50.0%)	6 (30.0%)	9 (45.0%)

### Irradiation promoted the activation of oxidative stress and PI3K-AKT signal pathway in DCS

**Figure 5:**
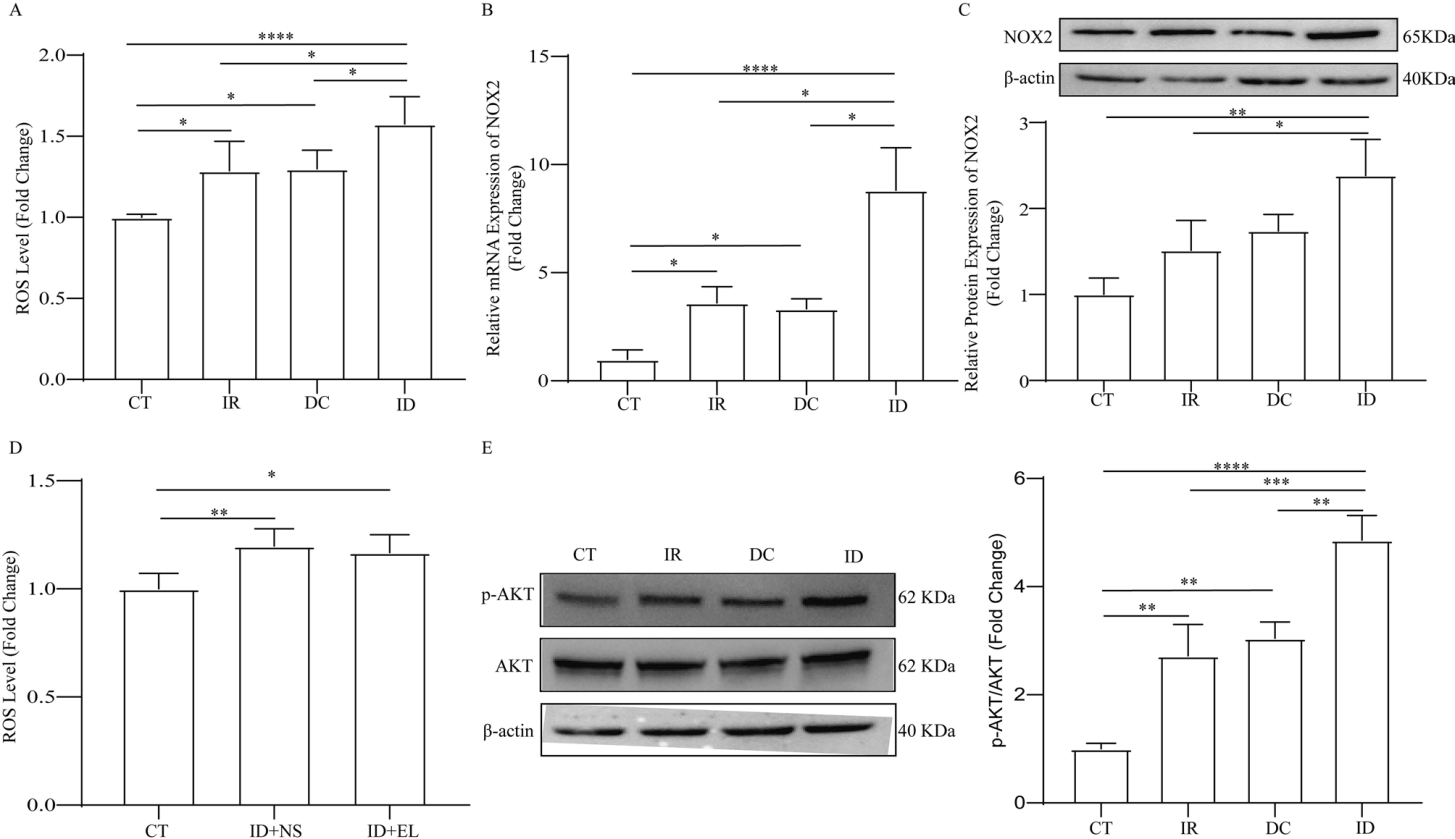
The impact of irradiation, decompression, and irradiation plus decompression injury on oxidative stress and PI3K-AKT signal pathway. The ROS level (**A**), NOX2 mRNA (**B**) and protein level (**C**) after irradiation, decompression, and irradiation plus decompression injury. The ROS level after irradiation plus decompression injury with or without enalapril treatment (**D**). Representative Western blot bands of p-AKT and AKT (left) and densitometric analysis of the p-AKT/AKT (right) in each group (**E**). The Values are mean ± SE; n = 5 in each group of (**A**), (**B**), and (**D**); n = 3 in each group of (**C**) and (**E**). *P < 0.05, **P < 0.01; ***P < 0.001; ****P < 0.0001.

We further detected the mechanism underlying the activated Ang II system in irradiation plus decompression injury. Both irradiation and decompression treatments increased the production of ROS compared with the CT group (Fig. 5A). And we found a significantly higher ROS level in the ID group than in other groups, showing the activation of oxidative stress in the lungs of rats undergoing irradiation plus decompression injury. Mechanically, the mRNA level and protein expression of NOX2, the critical enzyme of ROS synthesis, was significantly elevated after exposure to irradiation and decompression (Fig. 5B, C, S1). However, pre-treatment with enalapril made no difference to the ROS generated by irradiation plus decompression injury (Fig. 5D). Notably, the phosphorylation of AKT, the critical indicator of the PI3K-AKT signal pathway activation, was significantly elevated in the IR and DC groups, while it was significantly higher in the ID group than in other groups (Fig. 5E, S1).

## Discussion

The major findings of this study are as follows: (1) pre-exposure to radiation significantly exacerbated lung lesion and disease outcomes of DCS; (2) up-regulation of ACE expression and Ang II level was responsible for the damaging effect of predisposing irradiation on DCS; (3) Oxidative stress and PI3K/AKT signaling pathway activation in pulmonary tissue were enhanced in the injury of irradiation plus decompression. Based on these results, we conclude that pre-exposure to radiation elicits oxidative stress and activation of the PI3K/AKT signaling pathway, contributing to angiotensin system activation, exacerbated lung damage, and increased risk of DCS.

Nuclear submarines are extremely powerful. For nearly half a century, however, nuclear submarine accidents have been frequent around the world^30^. In the event of an accident, the consequences could be particularly severe, not only causing casualties and radioactive leaks but also potentially endangering the safety of the ship and even causing radioactive contamination of the marine environment^30,31^. The accident of the nuclear submarine Kursk is a real case of the dangers of nuclear submarine accidents. In such accidents, only those who have completed a rapid surfacing escape in the radioactive environment can be saved. However, the occurrence of DCS will be inevitable in this life-saving progress^1^. Although predecessors have advanced a lot in the field of DCS prophylaxis and treatment, the pathophysiological change in DCS under more complicated situation are still to be worked out. In the present study, we aimed to identify the impact of a certain dose of radiation exposure on the safety of fast buoyancy ascent escape and to explore the pathophysiological characteristics of the injury of irradiation plus decompression. High-dose irradiation exposure causes lethal damage, which still lacks effective treatment at present^32,33^. Hence, it is more meaningful to explore the prevention and treatment of DCS after non-lethal dose irradiation. Therefore, we established an animal model to simulate the injury of moderate dose radiation and followed decompression. In the present study, 4 Gy with a dose rate of 0.65 Gy/min irradiation was carried out, as it could lead to several characteristic irradiation pathophysiological events and intermediate prognosis without consequences of severe radiation damage complicated with trauma to many tissues and organs^34,35^. Inconsistent with previous studies, we found no severe organ damage or death in the IR group. Decompression treatment was carried out according to previous studies^36,37^, which led to 10% mortality in our study. Notably, we found 50% mortality in the ID group, which is significantly higher than in single-exposure groups. These results suggested that pre-exposure to radiation, even mild type, could significantly increase the mortality of DCS.

To explore the mechanism underlying the exacerbated DSC after irradiation, we examined whether the critical pathophysiological events in the DCS could be affected by irradiation exposure. In the DCS, rapid reduction of ambient pressure results in the disequilibrium between the alveolar and tissue nitrogen^1^. Excess nitrogen could be discharged into the pulmonary alveoli, while supersaturated gas forms bubbles and causes vascular damage^5^. Hence, we explored whether pre-exposure to radiation could affect lung function, exacerbate decompression-induced lung lesions, and increase the risk of DCS. Consistent with previous studies, decompression damage to an interstitium was found in the histopathological section of lung tissue^38,39^. After decompression treatment, parenchymal and vascular changes were observed, including pulmonary artery damage, diffuse alveolar damage, and edema. As reported, radiation exposure could also do harm to the pulmonary structure^10^. In previous studies, 15 Gy irradiation was the lowest dose of irradiation that could induce acute lung injury^40^. A lower dose of irradiation was found to have a chronic impact on the lungs. For instance, 5 Gy irradiation induced perivascular edema and pulmonary fibrosis at 4–8 weeks after exposure^41^. However, whether mild irradiation provokes acute changes in molecular events, like inflammation and oxidative stress is unclear. In the present study, we treated the rats with mild radiation and examined the lung injury at the early stage. Mild irradiation only induced changes in several indicators of inflammation, like IL-1β, IL-6, and TNF-α, while the pathological change in the lungs was not significant. Interestingly, irradiation made little difference, yet it significantly changed the outcomes of DCS. As indicated by the histopathological section and lung injury score, irradiation and followed decompression treatment substantially impacted pulmonary structure, which is much more severe than in the decompression-only group. The changes in the histopathological lesion of the lungs were correlated with the mortality in each group. Taken together, the mild irradiation had a minor effect on the lung structure but substantially impacted lung lesions and mortality of DCS. These results validated our hypothesis that pre-exposure to radiation affects lung function and exacerbates pulmonary vessel damage, the critical pathophysiological events caused by decompression, increasing the risk of DCS.

We further explored the critical molecular events underlying the exacerbated DCS after radiation exposure. The histopathology provided evidence. In irradiation plus decompression injury, the most substantial pathological change was observed in the pulmonary interstitium, including interstitium edema and inflammatory cell accumulation. These pathologies suggested that the vascular bed was mainly affected, and vasoactive substances may be involved in the pathophysiological process of irradiation plus decompression injury. Ang II is one of the most critical vasoactive agents, which causes vasoconstriction and vasospasm through AT_1_R^16^. Besides, Ang II could influence all the stages of the inflammatory response, including increased vascular permeability, leukocyte recruitment, and activation of tissue-repair processes^15^. Moreover, Ang II plays an essential role in radiation damage and DCS, respectively^18,19^. This interpretation naturally leads to a hypothesis that pre-exposure to irradiation promoted Ang II's vasoactive and pro-inflammatory effects and exacerbated vessel damage in DCS. In irradiation injury, Ang II has been demonstrated to contribute to radiation-induced chronic organ fibrosis and activation of several pathogenic signal molecules through its pro-inflammatory property^18^. In the present study, we only found the up-regulation of AT_1_R expression in the early stage of mild irradiation. Moreover, in DCS patients, all kinds of post-dive damages, like altered vascular permeability, platelet aggregation, and inflammation, could be mediated by Ang II. Consistent with previous results^19,21^, we also found a significant increase in Ang II level, ACE expression, and AT_1_R expression after decompression exposure. Notably, the activation of the angiotensin system in DCS was significantly promoted by irradiation pre-exposure. After the exposure to irradiation plus decompression, the Ang II level, ACE expression, ACE enzyme activity, and AT_1_R expression were all significantly elevated. These results suggested that either radiation or decompression laid limited effects on Ang II, while the exposure of irradiation plus decompression activated the angiotensin system profoundly, in the aspect of both synthesis and downstream effect. Irradiation exposure laid no impact on Ang II synthesis, hence, the up-regulation of ACE expression and Ang II level can not simply be explained by the effect of pile up. To better understand the activation of the angiotensin system in the irradiation plus decompression injury, we carried out the pharmacological intervention with both ACEI (enalapril) and AT1R inhibitor (losartan). Interestingly, as a result, the ACEI treatment showed significantly better protective effects against irradiation plus decompression. As a possible explanation for such results, in the pulmonary vessels, Ang II exerts its diverse downstream biological effects through multiple receptor pathways, including AT1R, AT2R, and Tie receptors^42–44^. Thus, interfering with Ang II production at its source and reducing the level of Ang II may be more effective than blocking one of its receptor pathways. Consequently, we further detected the role of elevated Ang II synthesis in the exacerbated DCS after irradiation with enalapril treatment. Rats pre-treated with enalapril showed significantly lower mortality undergoing irradiation plus decompression injury. Moreover, the pulmonary histopathology, w/d ratio, and inflammation indicators were all alleviated by enalapril treatment after irradiation plus decompression injury exposure. These results demonstrated that the increased Ang II level and enhanced Ang II synthesis is the vital mechanism underlying the impact of irradiation on DCS. In future research, the effect and effectiveness of ACEI in treating the injury of radiation plus decompression are worth further investigating.

Irradiation and followed decompression treatment significantly increased the Ang II level, in which the up-regulated ACE expression and enzyme activity play a pivotal role. However, through what mechanism radiation and decompression enhanced the ACE expression remained unclear. In the present manuscript, we detected the level of ROS, the critical pathophysiological mediators leading to vascular damage^45,46^, and found that the content of ROS was significantly increased in the irradiation, decompression, and irradiation plus decompression group, while the enalapril treatment did not significantly reduce the ROS level after irradiation plus decompression. Some evidence can support this finding. Ionizing radiation can directly break up the respiratory chain of mitochondria, producing ROS and reducing antioxidant capacity^47,48^. In DCS, bubbles and endothelial microparticles mediate endothelial dysfunction by the production of ROS^49,50^. Thus, in the disease model of irradiation plus decompression, the elevated level of ROS may largely be attributed to the biological events mentioned above, rather than the activated angiotensin system. Furthermore, this result suggested that oxidative stress may serve as the downstream mechanism of radiation and decompression exposure, yet the upstream mechanism of the angiotensin system activation. The increased oxidative stress after radiation was validated in our study, showing the up-regulation of NOX expression and ROS production. Similar results were observed in the decompression group. Moreover, the results also suggested that the use of antioxidants as a potential treatment deserves future research. Elevated oxidative stress could damage biological macromolecules and trigger a series of molecular signaling pathways^51,52^. Among them, PI3K/AKT signal pathway was reported to play a critical role in regulating ACE expression^53,54^. In lung tissue, we found that the phosphorylation of AKT was significantly elevated in the irradiation plus decompression injury group. At the same time, it showed no difference in the single-exposure groups compared with the blank control. Notably, the activation of the PI3K/AKT signal pathway showed the same trend as the mRNA level of ACE in the IR, DC, and ID groups, which indicates the close relationship between the PI3K/AKT pathway and ACE expression. One limitation of the study is that in vitro experiments were not further carried out to explore the effect of the PI3K/AKT signal pathway on ACE expression, as there is currently no suitable method to assess the impact of decompression on cultured cells. Future studies are needed to further demonstrate not only the role of the PI3K/AKT signal pathway in the regulation of ACE expression but also to explore the role of the PI3K/AKT signal pathway downstream of Ang II, as it is an important member of the Ang II downstream signaling pathway network. This will be helpful in exploring the role of the PI3K/AKT signal pathway in the irradiation exacerbated decompression injury.

In conclusion, this study demonstrates that predisposing irradiation exposure induces elevated oxidative stress and activates the PI3K/AKT signaling pathway, eliciting enhancement of Ang II synthesis and downstream effects, which then exacerbates lung injury in subsequent decompression exposure and leads to the aggravation of DCS. The present study pioneers the evaluation of the impact of irradiation on the safety of fast buoyancy ascent escape and explores the underlying mechanism, suggesting the pulmonary vessel is the critical target to increased sensitivity of decompression sickness after predisposing radiation exposure and highlighting the pivotal role of Ang II. Our study also suggested that the ACEI treatment may serve as a potential prophylaxis and treatment for such accidents, while exploration of the effective and practical pharmacological treatment is still necessary in future research (Supplementary Fig. S1).

## Supplementary Information

Supplementary Figure S1.

## Data Availability

The datasets generated during and/or analyzed during the current study are available from the corresponding author upon reasonable request.

## Abbreviations

DCS: Decompression sickness
AGE: Arterial gas embolism
Ang II: Angiotensin II
ACE: Angiotensin-converting enzyme
SD: Sprague–Dawley
ELISA: Enzyme-linked immunosorbent assay
IL: Interleukin
ROS: Reactive oxygen species
H&E: Hematoxylin and eosin
W/D: Wet-to-dry ratio
BALF: Bronchoalveolar lavage fluid
ACEI: Angiotensin-converting enzyme inhibitor
AT_1_R: Angiotensin 1 receptor
