# Supplementary figures and images for: Effect of angiotensin II on irradiation exacerbated decompression sickness
Source: Sci Rep. 2023 Jul 19;13:11659. doi: 10.1038/s41598-023-38752-z (PMC10356789; doi:10.1038/s41598-023-38752-z)

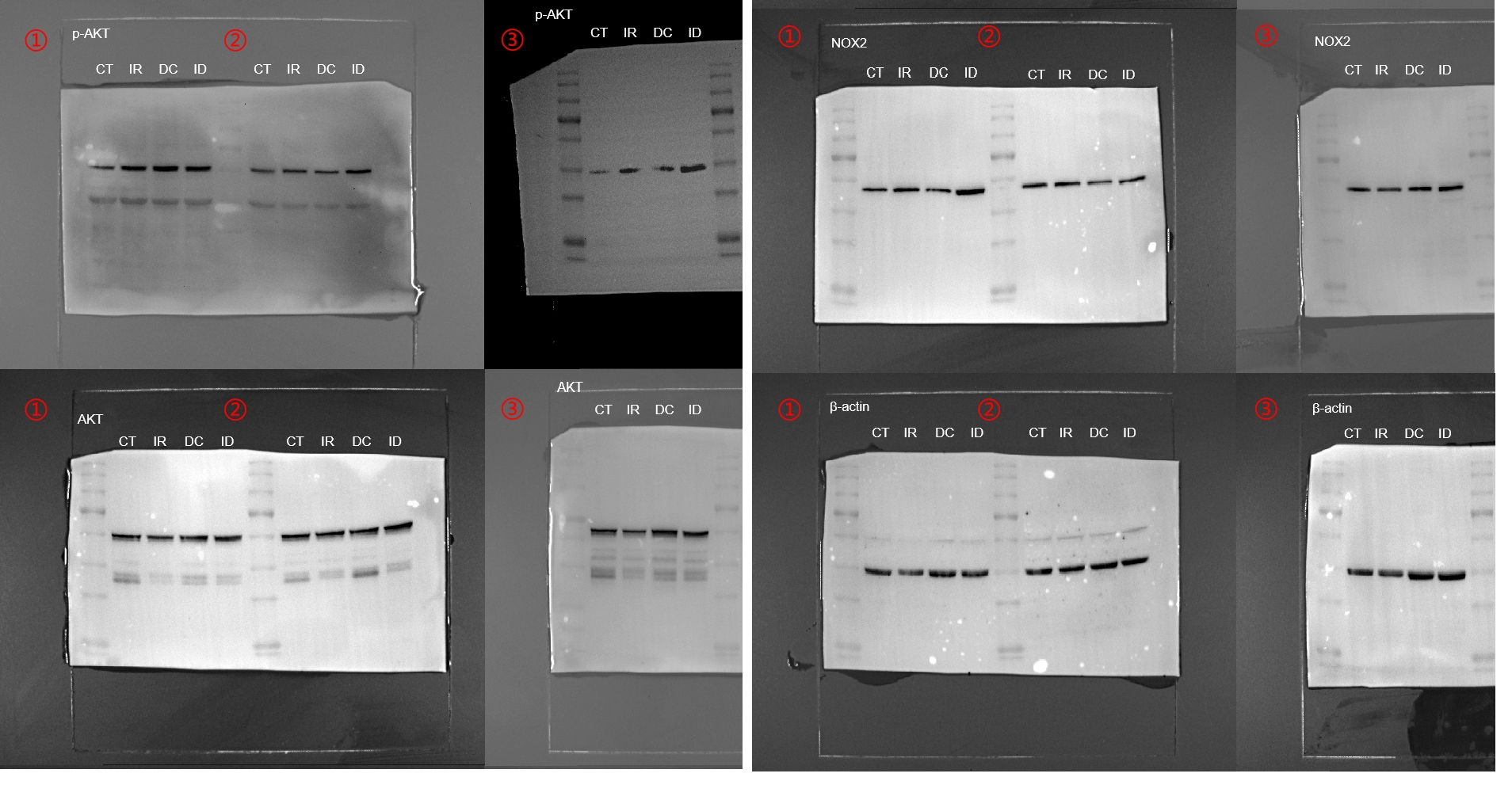


Figure S1: The original and unprocessed image of gels and blots.

Supplement: Supplementary file 1 — Supplementary Figure S1. [file 41598_2023_38752_MOESM1_ESM.docx]
